# Evaluating Clinically Directed Continuous Positive Airway Pressure to High Flow Nasal Cannula Transitions in Stable Preterm Infants Using Electrical Impedance Tomography: A Prospective, Observational Study

**DOI:** 10.1002/ppul.71328

**Published:** 2025-10-15

**Authors:** David M. Rub, Natalie Napolitano, Francis Simmons, Rachel Mackenzie, Kelle Matthews, Elizabeth E. Foglia, Howard B. Panitch

**Affiliations:** ^1^ Department of Pediatrics University of Pennsylvania Perelman School of Medicine Philadelphia Pennsylvania USA; ^2^ Division of Neonatology Children′s Hospital of Philadelphia Philadelphia Pennsylvania USA; ^3^ Department of Respiratory Care Children′s Hospital of Philadelphia Philadelphia Pennsylvania USA; ^4^ Division of Pulmonary and Sleep Medicine Children′s Hospital of Philadelphia Philadelphia Pennsylvania USA

## Abstract

**Objective:**

To assess lung aeration changes during clinically directed transitions from CPAP to HFNC in preterm infants using Electrical Impedance Tomography (EIT).

**Design:**

Prospective, observational study.

**Setting:**

Single‐center, Level IV Neonatal Intensive Care Unit.

**Patients:**

Infants born < 32 weeks gestational age (GA) undergoing a clinically indicated transition from CPAP to HFNC following ≥ 2 weeks of respiratory support.

**Interventions:**

EIT data were recorded for 30–60 min before and after transition.

**Main Outcome Measures:**

The primary outcome was change in end‐expiratory lung impedance (ΔEELI). Infants were followed for 7 days following transition to assess for transition failure.

**Results:**

From 15 subjects, 4257 total breaths were analyzed. No significant difference in %∆EELI was found between HFNC and CPAP (Median ∆: –1.0%; IQR –3.6% to 6.0%; *p* = 0.78). The largest %∆EELI decrease (–9.8%) occurred in the subject who failed transition.

**Conclusions:**

Transitioning from CPAP to HFNC did not consistently decrease lung aeration in stable preterm infants. In the infant who failed transition, a distinct respiratory pattern was observed using EIT, characterized by a decrease in EELI and frequent recruitment breaths. These findings suggest better methods are needed to individualize and titrate respiratory support at the bedside for preterm infants.

## 1 Introduction

Nasal continuous positive airway pressure (CPAP) is the most common form of noninvasive respiratory support used to help establish and maintain end‐expiratory lung volume (EELV) in preterm neonates. Increasingly, heated, humidified, high flow nasal cannula (HFNC) is being used as a weaning modality from CPAP for preterm infants requiring prolonged courses of noninvasive respiratory support [1]. HFNC provides a fixed rate of flow through narrow binasal prongs to relieve work of breathing by generating positive airway pressure and dead space carbon dioxide washout [2]. Reasons for transition to HFNC include reduction in nasal trauma, improved patient comfort, and parental preference [3]. Although CPAP to HFNC transitions are common, there is wide practice variability with respect to timing of transition and level of respiratory support before and after transition [4, 5].

A key contributor to this variability is the inability to equate the support delivered via nasal CPAP, set in cmH_2_O to that delivered by HFNC, in liters per minute (L/min). Flow rate, nasal cannula diameter, nares diameter, and mouth leak have all been shown to affect the distending pressure generated by HFNC [6, 7]. The degree to which HFNC maintains the EELV established by CPAP in weaning neonates is not known. Our neonatal intensive care unit (NICU) does not currently standardize the practice of CPAP to HFNC transitions.

Given the variability in both patient parameters and provider practice, we posited that successful transitions from CPAP to HFNC in preterm infants would be characterized by maintenance of EELV. Furthermore, we hypothesized that infants whose EELV decreased during transition would be at higher risk of transition failure. In this study, we used electrical impedance tomography (EIT) to assess changes in EELV associated with clinically directed transitions from nasal CPAP to HFNC in the NICU.

## 2 Methods

### 2.1 Study Design

The CPAP to HFNC EIT Observational Study (CHEITOS) was a prospective, observational study performed at the Children's Hospital of Philadelphia Neonatal/Infant Intensive Care Unit. The study was approved by the Children's Hospital of Philadelphia IRB. Parental informed consent was obtained for all subjects included in the study.

### 2.2 Population

Infants born < 32 weeks' gestational age (GA) were eligible if they received at least 2 weeks of continuous invasive or noninvasive respiratory support, were less than 50 weeks' corrected gestational age (CGA) at time of study, and had a predetermined clinical plan to transition from CPAP to HFNC. Infants with major congenital anomalies or chest wall abnormalities precluding EIT belt placement were excluded from the study. Patients were excluded following enrollment if the clinical team initiated systemic steroids, diuretics, albuterol, antibiotics, or acute fluid resuscitation within 48 h of transition.

Timing of transition, CPAP settings before transition, and HFNC settings following transition were independently determined by the clinical team. Although no standardized weaning protocol exists, infants in our unit are typically supported by 5–6 cmH_2_O CPAP and low F_I_O_2_ (0.21–0.25) before transition to HFNC. CPAP was delivered through the V500 (Draeger, Lubeck, Germany) using the Babyflow Plus nasal mask (Draeger, Lubeck, Germany). HFNC was delivered either through the Optiflow Jr. (Fisher & Paykel, Aukland, New Zealand) or the Precision Flow system (Vapotherm, Exeter, New Hampshire, USA).

### 2.3 Study Procedure

Approximately 1 h before transition, a LuMon EIT belt (SenTec AG, Landquart, Switzerland) containing 32 electrodes was placed around the supine infant's torso just inferior to the nipple line. Exact timing was coordinated with nursing and respiratory therapy teams based on availability and patient care needs. The LuMon EIT system (SenTec AG, Landquart, Switzerland) was used to record impedance data continuously at a frame rate of 51 Hz. Following a 30–60 min recording period on CPAP, the infant was transitioned to HFNC by the respiratory therapy team. EIT data were continuously recorded during transition and following transition to HFNC for an additional 30–60 min.

### 2.4 Data Analysis

A minimum of 100 artifact‐free breaths per subject before and after transition was extracted and analyzed using iBex (version 1.5; SenTec AG). For the post transition phase, breaths were extracted following equilibration of EELI. EIT signals were reconstructed within lung regions defined by the vendor‐provided human model chest atlas. To minimize lung contouring bias, symmetric lung contours were used for all analyses. Basic low‐pass filtering was used to reduce heart rate artifact and enhance signal quality when applicable.

### 2.5 Outcome

The primary outcome of this study was percent change in end‐expiratory lung impedance (%∆EELI) before and after transition from CPAP to HFNC. Secondary exploratory outcomes, as described in Table S2, included percent change in end‐inspiratory lung impedance (%∆EILI), global and regional tidal volume impedance (TVI), vertical and horizontal center of ventilation (CoV(v) and CoV(h), respectively), and silent space percentage (SS%). Individual breath EELI was referenced to the mean EELI value for each subject during CPAP to calculate a “Normalized EELI” [8]. Subject respiratory status and weight gain were monitored for 1 week following transition. “Failure” of transition was defined as a clinical decision to revert to CPAP, nasal intermittent mechanical ventilation (NIMV) or invasive mechanical ventilation (IMV) within 7 days of transition. Because noninvasive transition failure is not well characterized in the literature, we selected a 7‐day cutoff by extrapolating from the extubation failure evidence base [9]. This approach aimed to optimize the identification of respiratory‐related failures while minimizing the inclusion of failures due to nonrespiratory causes.

### 2.6 Statistical Analysis

Baseline and demographic characteristics were summarized using standard descriptive statistics. Paired inferential analysis of primary and secondary outcomes before and after transition to HFNC was performed using the Wilcoxon signed‐rank test. Original sample size calculations before enrollment were based on independent groups. An updated calculation that was conducted after enrollment started appropriately accounted for the paired means design. The calculation revealed a sample of 15 patients would provide 80% power to detect a 10% difference in EELI within subjects, assuming a standard deviation of 0.1–0.15 (Figure S1). The 10% threshold was derived from prior study in preterm infants using EIT to evaluate changes in EELI following extubation [8].

Additionally, a post hoc analysis was performed to evaluate the effect of the HFNC device (Fisher & Paykel Optiflow Jr. vs. Vapotherm Precision Flow) on %ΔEELI using Wilcoxon rank‐sum test.

All statistical analyses and power calculations were performed using STATA v18.0 (STATA Corp, TX, USA). *p* < 0.05 were considered statistically significant.

## 3 Results

**Figure 1:**
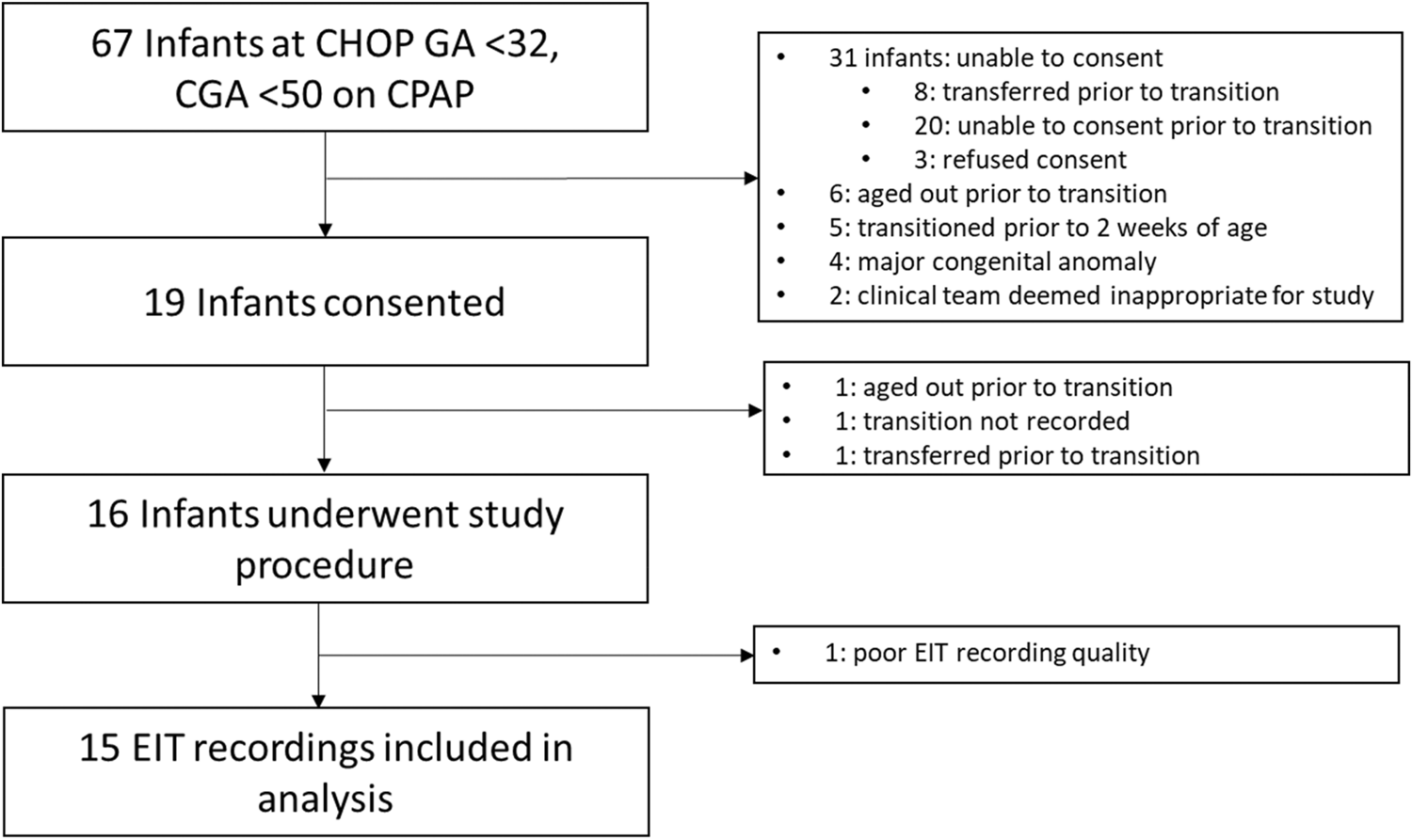
Flow diagram representing patients consented and included or excluded in the study. Reasons for exclusion included at each stage of enrollment.

Nineteen subjects were consented between February 2023 and August 2024. Of the 16 subjects who completed the study, 15 subjects had recordings of suitable quality and were included in the analysis (Figure 1). The median (IQR) gestational age was 27.6 (25.6–29.4) weeks and 11 (73%) were female. At time of transition, the median (IQR) corrected gestational age was 35.1 (32.6–40.3) weeks. The median (IQR) CPAP level and fiO2 at time of transition was 5 (5–6) cmH_2_O and 0.21 (0.21–0.23). The median (IQR) HFNC level immediately post‐transition was 5 (5–6) L/min or 3.0 (2.0–3.6) L/kg/min when adjusted for weight. Complete summary demographic and respiratory support data are included in Table 1. Among the 15 subjects, 1 met transition failure criteria by the end of the study period, and 1 was unable to be followed for the entire 7‐day posttransition period (Table S1). All infants tolerated the EIT recording during the study period.

**Table 1:** Summary demographics.

*N* = 15	
Gestational age at birth, weeks	27.6 (25.6, 29.4)^a^
Birthweight, g	750 (680, 1055)
Female, *n* (%)	11 (73%)
C‐section, *n* (%)	10 (67%)
Antenatal steroids,^b^ *n* (%)	11 (73%)
History of surfactant, *n* (%)	11 (73%)
History of invasive mechanical ventilation, *n* (%)	12 (80%)
Duration of invasive mechanical ventilation, days	12 (5, 71)
Duration of noninvasive ventilation, days	34 (21, 56)
Stable CPAP before transition,^c^ days	6 (3, 8)
Postnatal age at time of transition, days	52 (26, 85)
Corrected gestational age at time of transition, weeks	35.1 (32.6, 40.3)
Weight at time of transition, g	1865 (1400, 2485)
CPAP level immediately before transition, cmH_2_O	5 (5, 6)
Baseline FiO_2_ before transition	0.21 (0.21–0.23)
HFNC level immediately after transition, L/min	5 (5, 6)
Weight adjusted HFNC level, L/kg/min	3.0 (2.0, 3.6)

a Data presented as medians with interquartile ranges unless otherwise specified.

b Antenatal steroid exposure includes only complete courses as documented in the electronic medical record.

c Represents the number of days on CPAP at the support level immediately before transitioning to HFNC.

A total of 4257 breaths were included in the primary analysis. A median of 300 breaths were analyzed per subject (interquartile range [IQR] 219–340). The median number of breaths analyzed on CPAP and HFNC support were 123 (IQR 114–137) and 115 (IQR 100–204), respectively. Thirty‐three breaths with missing region of interest data or negative tidal volume impedance were treated as artifact and excluded from the analysis.

Analysis of the primary objective revealed no statistically significant difference in the normalized EELI between infants supported by HFNC compared to CPAP (median difference −1.0%; IQR −3.6% to 6.0%). Additionally, there were no statistically significant differences in EILI, TVI, i‐time or in the reported measures of heterogeneity (Table 2). No differences in the primary outcome were observed between HFNC device type.

**Table 2:** CPAP vs. HFNC change in EIT variables^a^.

	CPAP	HFNC	*p* value^b^
Normalized^c^ EELI	1 (1–1)	0.99 (0.97–1.06)	0.78
Normalized EILI	1.02 (1.02–1.03)	1.01 (0.994–1.06)	0.91
Normalized TVI	0.022 (0.019–0.028)	0.022 (0.017–0.029)	0.49
i‐Time (seconds)	0.43 (0.41–0.52)	0.49 (0.39–0.54)	0.73
Measures of heterogeneity			
CoV–VD (%)	58.1 (54.4–60.2)	57.0 (55.0–60.8)	0.82
CoV–RL (%)	45.1 (42.8–48.1)	45.1 (43.2–48.1)	0.28
Regions of interest			
Ventral	13.2 (11.1–17.9)	14.1 (10.6–16.1)	0.82
Central‐ventral	25.8 (24.7–28.4)	27.1 (24.5–28.5)	0.36
Central‐dorsal	35.1 (34.2–36.6)	34.2 (33.7–37.1)	0.57
Dorsal	26.5 (21.0–28.3)	23.8 (22.1–28.0)	0.65

^a^ End‐expiratory lung impedance (EELI); end‐inspiratory lung impedance (EILI); tidal volume impedance (TVI); inspiratory time (i‐time); center of ventilation (CoV); ventral‐dorsal (VD); right‐left (RL); all values reported as median (IQR) unless otherwise specified.

^b^ Wilcoxon signed‐rank test used for comparison before and after transition from CPAP to HFNC

^c^ EELI, EILI, TVI all normalized using baseline mean EELI on CPAP as reference.

**Figure 2:**
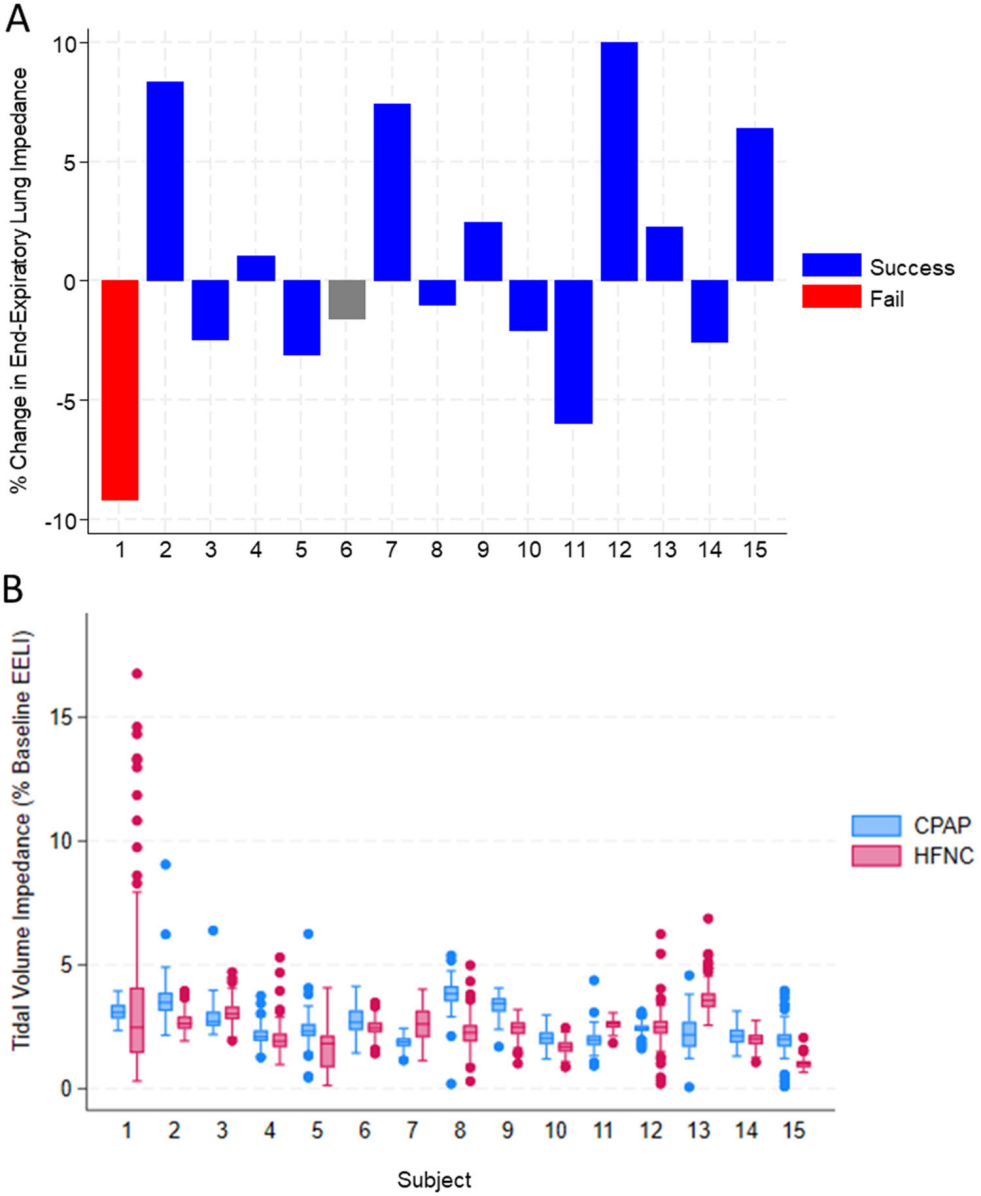
(A) Percentage change in end‐expiratory lung impedance (∆EELI) for each subject following the transition from CPAP to HFNC. Each bar represents an individual subject; blue bars indicate a successful transition, while red bars denote a transition failure. Transition failure defined as reversion to CPAP, nasal intermittent mandatory ventilation (NIMV) or invasive mechanical ventilation (IMV) within 7 days of transition. Subject 1 was the only subject meeting the criteria for transition failure. There was incomplete follow‐up data for Subject 6. (B) Distribution of tidal volume impedance (TVI) as a percentage of baseline EELI for each subject during CPAP (blue) and HFNC (red). Box plots display the median, interquartile range, and outliers, with individual points representing breaths. Subject 1 exhibited elevated TVI variability and presence of frequent, recruitment breaths (TVI > 99th percentile) on HFNC. [Color figure can be viewed at wileyonlinelibrary.com]

**Figure 3:**
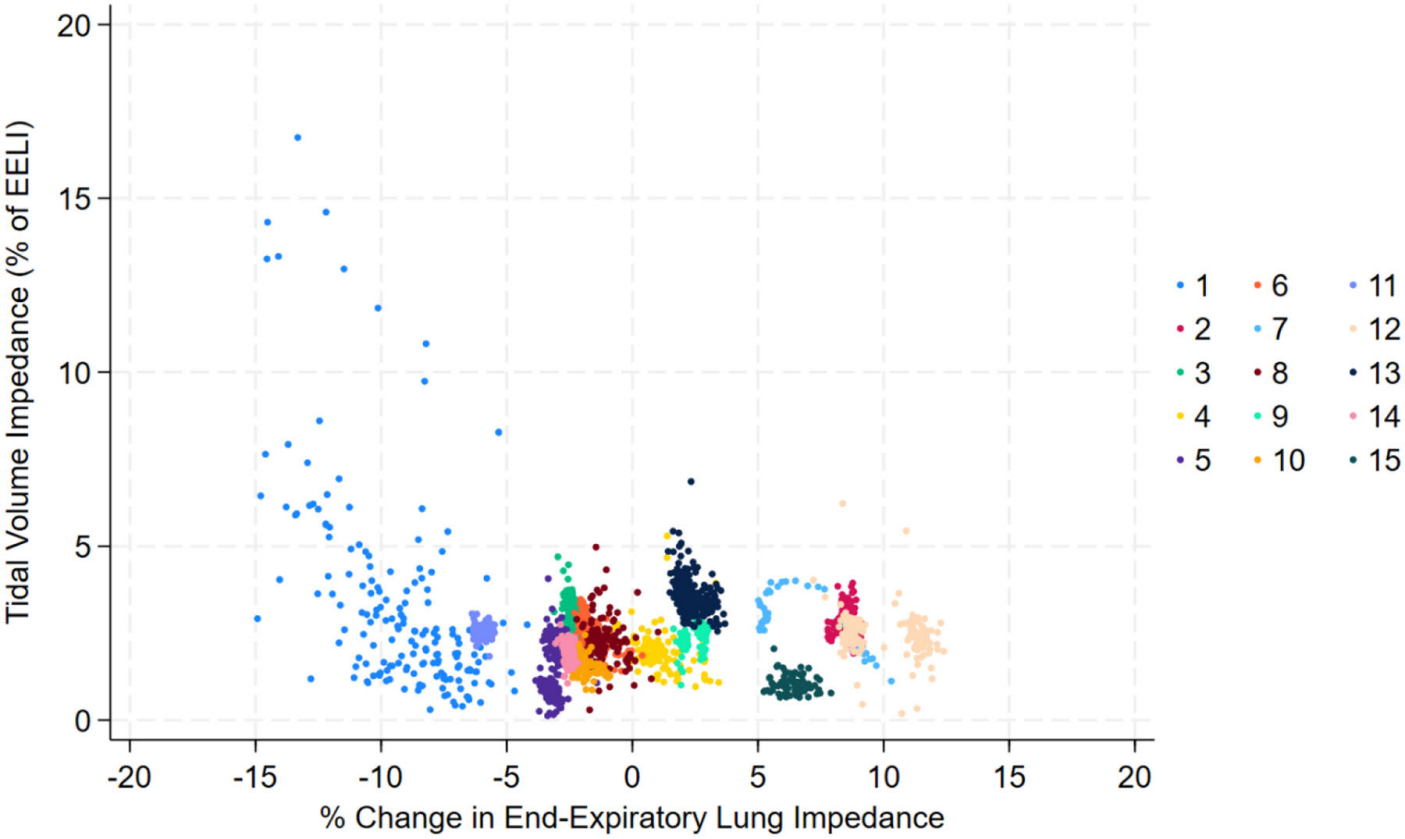
Scatter plot illustrating the relationship between tidal volume impedance (TVI) as a percentage of EELI and percent change in end‐expiratory lung impedance (EELI) across individual breaths for each subject following the transition from CPAP to HFNC. Each color represents a different subject, as indicated in the legend. [Color figure can be viewed at wileyonlinelibrary.com]

When analyzed individually, 4 of the 15 subjects experienced a greater than 5% increase in EELI following transition to HFNC, while 2 subjects experienced a greater than 5% decrease (Figure 2A). Notably the greatest decrease in EELI (−9.2%) occurred in the subject who failed transition (Figure 2A, Subject 1). This subject also exhibited a distinct pattern of large tidal volume impedance breaths, as depicted in Figure 2B. The relationship between TVI and %∆EELI for all breaths across subjects following transition to HFNC is depicted in Figure 3.

## 4 Discussion

In this prospective, observational study of preterm infants requiring prolonged respiratory support, we evaluated changes in breathing pattern and maintenance of FRC following transition from CPAP to HFNC using EIT. Notably, we found that transitioning from CPAP to HFNC did not consistently decrease EELV, as measured by %∆EELI.

In the NICU, it is commonly believed that a transition from CPAP to HFNC results in a wean in respiratory support [10, 11]. This assumption is extrapolated from clinical trials comparing CPAP and HFNC as primary or post‐extubation support in the setting of respiratory distress syndrome (RDS) [4, 5]. Our findings directly confront this assumption. We did not find a consistent, significant reduction in EELV when transitioning from CPAP to HFNC. In fact, 25% of the infants experienced a significant increase in EELV following transition to HFNC. These findings suggest there may be a cohort of infants who would benefit from earlier transition to HFNC, resulting in improved patient comfort and decreased risk of nasal trauma [3]. An earlier transition strategy is further supported by the remarkably low failure rate in our cohort of 6.7%.

These findings directly contrast with a recent study by Buchler and colleagues, which also examined CPAP to HFNC transitions using EIT [12]. Unlike our study, Buchler and colleagues concluded that transitions to HFNC are likely to result in reduced end‐expiratory lung volume [12]. However, key differences in study design may account for these divergent results. One likely factor behind this discrepancy is that our study population was older, with a median CGA of 35 weeks, compared to a median CGA of 32 weeks in their population. Substantial changes in chest wall stabilization occur between 30 and 36 weeks CGA due to improved rib ossification and intercostal muscle strengthening [13]. Chest wall maturity plays a crucial role in maintaining end‐expiratory lung volume and may account for the observed differences in outcomes between the two studies. Additionally, in our study, stability on CPAP was defined by the clinical team rather than strictly by research protocol, which may have led to longer periods on CPAP before transitioning to HFNC. Finally, in our unit infants remained supine, while in Buchlerand colleagues study, infants were positioned prone following the transition per their unit protocol. Prone positioning is known to influence respiratory mechanics, which may have contributed to the differing outcomes observed [14, 15].

Two unique aspects of our study were our description of the respiratory pattern in the infant who failed transition and our reporting of weight‐based HFNC flow rates. While transition failure is well studied in the context of extubation, it remains undefined for preterm infants transitioning between noninvasive respiratory support modalities. For our study, we defined transition failure as reverting back to CPAP, nasal intermittent mechanical ventilation (NIMV) or invasive mechanical ventilation (IMV) within 7 days of HFNC initiation. The decision to revert to higher level of support was made entirely by the clinical team.

Surprisingly, only 1 subject met failure criteria. This subject reverted to CPAP after 6 days of HFNC therapy. As previously noted, this subject experienced the largest drop in EELI among all subjects (Figure 2A) and exhibited frequent, large tidal volume breaths (Figures 2B and 3). Preterm infants utilize a variety of strategies to maintain FRC including dynamic hyperinflation and laryngeal braking [16]. We believe the pattern displayed by this subject represented an overwhelmed respiratory system which required exceedingly large tidal volumes in addition to the aforementioned recruitment strategies. This process is likely to be energy intensive, limiting post‐natal growth, and injurious to the lungs through recurrent volutrauma [17, 18].

One of the difficulties in selecting HFNC flow rate is the inability to equate units of HFNC (L/min) to units of CPAP support (cmH_2_O). One strategy used to overcome this in pediatric practice is weight‐based HFNC flow rates (L/kg/min). When compared to a fixed flow rate strategy, weight‐based flow rates of 1.5–2 L/kg/min have been shown to reduce hospital length of stay and ICU admissions for children with bronchiolitis [19, 20]. Though we are cautious to extrapolate results across different patient populations, we do note that the median (IQR) weight‐based flow in our study, 3.0 (2.0–3.6) L/kg/min, was above the pediatric target for acute illness. While weight‐based flow rates were not associated with the outcomes measured in this study, reporting weight‐based flow rates may be useful for standardizing HFNC support levels and comparing results across future studies.

Strengths of this study include its prospective nature and the use of EIT to quantify changes objectively in regional and whole‐lung aeration. Additionally, its observational design allows us to draw conclusions regarding CPAP to HFNC transitions as intended by the clinicians. The heterogeneity introduced by this design enhances its generalizability and elucidates the limited tools available to clinicians when making decisions regarding respiratory support needs. Furthermore, this study adds to the growing body of literature using EIT to characterize the effects of noninvasive ventilation on preterm respiratory physiology [8, 21, 22, 23, 24].

Conversely, there are several limitations to consider. First, it was conducted at a single site, which may limit the generalizability of our findings, as it does not capture potential variability in the timing and respiratory support settings used at other NICUs. Additionally, our low failure rate may reflect selection bias, as time constraints during recruitment may have led to the enrollment of infants with greater stability on CPAP. Moreover, only one enrolled infant met transition failure criteria, which limited our ability to compare respiratory patterns between successful and failed cases. To address this, we included patient ID as a fixed effect in our regression model to account for patient‐level clustering.

## 5 Conclusion

In conclusion, we were able to demonstrate empirically that transitions from CPAP to HFNC do not consistently result in decreased end‐expiratory lung volumes during standard clinical practice. Moreover, we describe a unique respiratory pattern that may serve as an early predictor of HFNC therapy failure. Additional studies are needed to define CPAP to HFNC transition failure, evaluate the clinical importance of transition failure, and further explore the role of EIT in predicting transition failure.

## Author Contributions

**David M. Rub:** conceptualization, investigation, funding acquisition, writing – original draft, methodology, visualization, data curation. **Natalie Napolitano:** conceptualization, investigation, writing – review and editing, methodology. **Francis Simmons:** data curation, writing – review and editing. **Rachel Mackenzie:** data curation, writing – review and editing. **Kelle Matthews:** data curation, writing – review and editing. **Elizabeth E Foglia:** writing – review and editing, supervision, resources. **Howard B Panitch:** writing – review and editing, conceptualization, investigation, methodology, supervision, resources.

## Ethics Statement

The study was approved by the Children's Hospital of Philadelphia IRB (IRB #23‐020747). Parental informed consent was obtained for all subjects included in the study.

## Conflicts of Interest

N.N.—Research and/or consulting relationships with VERO‐Biotech, Actuated Medical, Drager, and Timpel. All arrangements and payment are through the Children′s Hospital of Philadelphia. The other authors declare no conflicts of interest.

## Supporting information


**Supplemental Figure 1:** Power Calculation. **Supplemental Table 1:** Clinical Characteristics and Outcome per Subject. **Supplementary Table 2:** Description of Electrical Impedance Tomography Measures.

## Data Availability

Study protocol and deidentified raw data are available upon request to corresponding author.
