# Supplementary material for: Evaluating Clinically Directed Continuous Positive Airway Pressure to High Flow Nasal Cannula Transitions in Stable Preterm Infants Using Electrical Impedance Tomography: A Prospective, Observational Study
Source: Pediatr Pulmonol. 2025 Oct 15;60(10):e71328. doi: 10.1002/ppul.71328 (PMC12522079; doi:10.1002/ppul.71328)
Supplement: Supplementary file 1 — Supplemental Figure 1: Power Calculation. Supplemental Table 1: Clinical Characteristics and Outcome per Subject. Supplementary Table 2: Description of Electrical Impedance Tomography Measures. [file PPUL-60-0-s001.docx]

**SUPPLEMENT**

**Supplemental Table 1: Clinical Characteristics and Outcome per Subject**

| Subject | Gestational Age (weeks) | Postnatal Age (days) | CGA (weeks) | CPAP Level (cmH2O) | HFNC Level (L/min) | Weight-Adjusted HFNC Level (L/kg/min) | Days on stable CPAP prior to transition | % Weight Change over 3 days | % Weight change over 7 days | Transition Outcome |
| --- | --- | --- | --- | --- | --- | --- | --- | --- | --- | --- |
| 1 | 27 | 83 | 39 | 6 | 5 | 2.4 | 6 | 0% | 3% | Failure |
| 2 | 26 | 85 | 38 | 6 | 5 | 2.2 | 2 | 6% | 9% | Success |
| 3 | 26 | 39 | 32 | 5 | 5 | 3.0 | 8 | 2% | 10% | Success |
| 4 | 29 | 40 | 35 | 5 | 4 | 1.9 | 2 | 7% | 9% | Success |
| 5 | 29 | 39 | 34 | 6 | 6 | 3.6 | 7 | 1% | 6% | Success |
| 6 | 23 | 52 | 30 | 5 | 5 | 4.3 | 6 | 3% | 8% | n/a** |
| 7 | 28 | 16 | 30 | 6 | 5 | 3.8 | 4 | 5% | 7% | Success |
| 8 | 29 | 81 | 41 | 5 | 5 | 2.0 | 11 | 4% | 9% | Success |
| 9 | 27 | 66 | 36 | 5 | 6 | 3.2 | 20 | 5% | 9% | Success |
| 10 | 24 | 138 | 44 | 5 | 5 | 1.4 | 6 | 2% | 6% | Success |
| 11 | 29 | 25 | 32 | 5 | 6 | 4.1 | 3 | -3% | -4% | Success |
| 12 | 31 | 14 | 33 | 5 | 5 | 3.6 | 3 | 2% | 14% | Success |
| 13 | 25 | 103 | 40 | 6 | 7 | 2.3 | 5 | 2% | 7% | Success |
| 14 | 30 | 26 | 33 | 6 | 4 | 3.1 | 2 | 10% | 17% | Success |
| 15 | 25 | 141 | 45 | 5 | 5 | 1.2 | 8 | 1% | 7% | Success |

*Failure defined as reversion to CPAP within 7 days of transition

**subject 6 underwent a surgical procedure within 7 days of transition, unable to assess outcome

| **Supplementary Table 2: Description of Electrical Impedance Tomography Measures** | |
| --- | --- |
| **EIT Measures (Abbreviation)** | **Description and Physiologic Interpretation** |
| Functional Measure of Ventilation |  |
| End-Expiratory Lung Impedance (EELI) | Minimum impedance value per measurement interval. Estimate of lung volume at end tidal expiration (functional residual capacity) |
| End-Inspiratory Lung Impedance (EILI) | Maximum impedance value per measurement interval. Estimate of lung volume at end tidal inspiration (functional residual capacity + tidal volume) |
| Tidal Volume Impedance (TVI) | Difference between EILI and EELI per measurement interval. Estimate of tidal volume. |
| Spatial Distribution of Ventilation |  |
| Silent Space Percentage (SS%) | Percentage of region of interest with less than 10% change in impedance during breathing. |
| Center of Ventilation (CoV) | Graphic coordinate representing weighted geometric center of ventilation. |
| Vertical (CoV(v)) | CoV measured along the ventral – dorsal axis. |
| Horizontal (CoV(h)) | CoV measured along the left – right axis. |

**Supplemental Figure 1: Power Calculation**

****Line graph depicting two-sample paired-means power calculation to detect a 10% difference in end-expiratorylung impedance (EELI) before and after transition across 3 different standard deviation estimates (0.05, 0.1, 0.15) with 80% power at a 0.05 significance level.
